# Assessment of sexual and reproductive access and use of menstrual products among Venezuelan migrant adult women at the Brazilian–Venezuelan border

**DOI:** 10.1016/j.jmh.2022.100097

**Published:** 2022-04-05

**Authors:** Leila Rocha, Rachel Soeiro, Noé Gomez, Maria Laura Costa, Fernanda G. Surita, Luis Bahamondes

**Affiliations:** Department of Obstetrics and Gynecology, University of Campinas Faculty of Medical Sciences, Campinas, SP, Brazil

**Keywords:** Migrant women, Venezuela, Reproductive health, Period poverty, Access, Modern contraceptives

## Abstract

**Objective:**

To describe the sociodemographic characteristics, access to sexual and reproductive health (SRH) care, including contraceptives and to assess menstrual poverty of migrant Venezuelan adult women of childbearing age at the northwestern border between Venezuela and Brazil.

**Methods:**

Cross-sectional study coordinated by the Department of Obstetrics and Gynecology, University of Campinas, Campinas, SP, Brazil, conducted in Boa Vista, Roraima between January 18 and 24, 2021. We invited women aged 18 to 49 years to participate. A semi-structured self-response questionnaire was applied. The survey covered issues relating to SRH services, knowledge, access, and use of SRH services for women. We also applied a questionnaire regarding access to and quality of hygiene kits and toilets, and an open-ended question on “*what does menstruation mean to you”*? We excluded illiterate women and those with amenorrhea, those who had undergone hysterectomy or tubal ligation, and those with partners who had undergone vasectomy.

**Findings:**

The age (mean ± SD) of the 177 respondent women was 28 ± 6.8 years, 32.2% reported that they had more than three children, 38.4% referred at least one unplanned pregnancy, and 52.5% of the women indicated an intention to become pregnant in the near future. Furthermore, 40 (29.8%) women sought a healthcare service because they wanted a contraceptive method; among them, 16 did not receive the contraceptive that they chose, and 15 women wanted to use a contraceptive implant. Regarding menstrual poverty, 64 women stated that the menstrual hygiene products provided by humanitarian organizations were not enough for their needs, and 44 women claimed being unable to wash their hands anytime they wanted to.

**Conclusions:**

The vulnerabilities of this cohort of Venezuelan migrant women in Brazil who lived mainly out of the official shelters further increase when they struggle with no knowledge of how to access SRH services, lack of provision of LARC methods, risk of unplanned pregnancy, and inappropriate access to menstrual hygiene products and sanitary services. There are several challenges to be overcome to ensure SRH care for migrant women in Brazil.

## Highlights

• Difficulties to access to sexual and reproductive services.
• Lack of access to contraceptives mainly LARCs.
• High rate of unplanned pregnancy.
• Low provision of menstrual products.
• Lack of access to sanitation facilities.

## 1 Introduction

Among the nearly 4.8 million Venezuelan migrants around the world who have left country due to the economic, political, and health crisis, almost 225,000 of these migrants are currently in Brazil in different migratory situations (UNHCR, 2020). In 2012, the Venezuelan health system went into crisis with the reduction of its capacity for care, which was aggravated in 2015 when a combination of factors, such as lack of public services, reduction of healthcare professionals, and lack of commodities and drugs, which results in the resurgence of controlled diseases, increased child mortality by 30.1% and maternal mortality by 65.4% (Page et al., 2019). Among other problems, there has been a lack of availability of contraceptive methods in the public sector since 2015, mainly long-acting reversible contraceptives (LARCs) (Page et al., 2019). This situation has mainly impacted on the low-income portion of society and has had profound consequences on women's sexual and reproductive health (SRH) and rights (Doocy *et al*., 2019).

Roraima is the Brazilian state which is the main gateway from Venezuela; one of the poorest Brazilian states, with 600,000 inhabitants and almost 70% living in the capital, Boa Vista. It was estimated that in 2021 the population of the state grew by 3.4% compared to 2020, motivated by migration. This growth increased the demand for health services, which were overwhelmed before the migration crisis and with the increasing crisis after the SARS-CoV-2 (COVID-19) pandemic (IBGE, 2021).

According to the Brazilian constitution, health is an obligation of the state and a right for all, and the Unified Health System (*Sistema Único de Saúde*, SUS) served almost 74% of the population, without distinction between nationals and legal and non-legal migrants. In Boa Vista there is only one public maternity and children's hospital, and they reported 288 deliveries by Venezuelan women in 2016, with an increase in subsequent years: 572 in 2017, 1,629 in 2018, 2,875 in 2019, and 2,300 in 2020, which represented, respectively, 3.4%, 6.6%, 16.4%, 26.1%, and 22.9% of the total births in the city (Brasil. Ministério da Saúde 2020). Our group conducted three rounds of visits to the Roraima state to interviewed women at reproductive health and adolescents to assess access to SRH care, violence against women, and menstrual poverty (Bahamondes *et al*., 2020; Makuch et al., 2021a, Makuch et al., 2021b; Soeiro et al., 2021a, Soeiro et al., 2021b). We found difficulties for Venezuelan migrants to access to SRH, among those who requested contraceptives, 63.3% reported that they were unable to obtain the contraceptive that they wished, and 50.6% reported that they were unable to obtain any contraceptives due to lack of availability (Bahamondes *et al*., 2020), and according to the perspective of these migrant women violence was part of everyday life (Makuch et al., 2021a, Makuch et al., 2021b). However, the interviews that we conducted previously and presented in previous reports (Bahamondes *et al*., 2020; Makuch et al., 2021a, Makuch et al., 2021b; Bahamondes *et al*, 2022) were performed with women living in the official shelters established by the United Nations High Commissioner for Refugees (UNHCR) and the Brazilian army and the other two reports (Soeiro et al., 2021a, Soeiro et al., 2021b) were conducted with interviews with adolescent girls.

These highlight the need for full cooperation among national and local authorities and charitable organizations, which are working in the territory to plan and enable the needed resources, thus guaranteeing social and economic rights together with SRH and rights is unfeasible without the necessary structural conditions that provide for women's survival. As this is the first time that Brazil has received a large migratory flow, data on the SRH of migrant women in the Amazon region are still scarce. Therefore, our objective was to describe the sociodemographic characteristics, access to sexual and reproductive health (SRH) care, including contraceptives and to assess menstrual poverty of migrant Venezuelan adult women of childbearing age at the northwestern border between Venezuela and Brazil.

## 2 Methods

We conducted a cross-sectional quantitative study coordinated by the Department of Obstetrics and Gynecology, Faculty of Medical Sciences, University of Campinas, Campinas, SP, Brazil. The university's ethics committee approved the study project (CAAE #204582219.0.0000.5404), and all participants signed an informed consent form prior to enrollment. The study was conducted in Boa Vista, Roraima between January 18 and 24, 2021, and two trained interviewers, a nurse and a physician (fluently in Spanish), conducted all face-to-face invitations to participate and provided a pre-tested semi-structured self-report questionnaire with open-ended and closed-ended questions, developed from the readiness assessment tools of the Interagency Working Group (IAWG) Minimum Initial Service Package (MISP) readiness on Reproductive Health in Crisis (Inter-Agency Working Group 2017). The survey covered a number of issues relating to SRH services, knowledge, access, and use of SRH services by women of reproductive age.

In addition, to assess menstrual poverty, we applied a questionnaire developed specifically for this study that was adapted from the Menstrual Practice Needs Scale (MPNS-36) (Hennegan *et al*., 2020) and translated into Spanish (native Venezuelan language) and validated previously. This questionnaire included a scale developed after a review of the literature on menstrual practices in low- and middle-income countries. It was tested in Uganda as a pilot survey and can be adapted to different ages and contexts. The questionnaire included multiple-choice questions on sociodemographic characteristics, access and quality of hygiene kits and toilets, and the open-ended question “*what does menstruation mean to you*?” Menstrual poverty or menstrual hygiene management (MHM) is an unmet aspect of SRH and was defined a lack of access to menstrual products, inadequate access to safe, clean, and private sanitation facilities, and adequate education (Soeiro *et al*., 2021a).

We included Venezuelan migrant women aged 18 to 49 years, and we excluded illiterate women (because the questionnaire was self-responded), those in amenorrhea, those who had undergone hysterectomy or tubal ligation, and those with partners who had undergone vasectomy. Participants did not receive any financial compensation.

Regarding public health facilities where it is possible to obtain SRH care, Boa Vista has 34 primary care health posts, each responsible for about 13,000 inhabitants, a maternity and child hospital, and two reference centers for women's health. Although the national Unified Health System offers contraceptives at no cost (medroxyprogesterone injection, once-a-month injectables, combined oral contraceptives [COC], progestin-oral contraceptives, TCu380A intrauterine device [Cu-IUD]), condoms, and emergency contraceptive pills (Brasil. Ministério da Saúde 2009), at the time of the study, the only contraceptives available in the primary care network were injections and COCs.

To describe the profile of the sample, frequency tables of the categorical variables were prepared, with absolute (n) and percentage (%) frequency values. A figure containing categorized responses was created, with the absolute numbers of responses in each category. We use the SAS (Analytics Software & Solutions) version 9.4 program. For the open question “*How is menstruation for you*?”, the answers were grouped by the frequency of the most used words and analyzed by similarity.

## 3 Results

Initially, our proposal was to invite women who lived in the shelters established by the UNHCR and the Brazilian army; however, due to the COVID-19 pandemic, we were unable to enter the shelters, and we decided to invite migrant women who came to a church (*Nossa Senhora da Consolata*) to receive food and other goods they needed and at the city bus station, in which the migrants have an informal shelter that receives some support from the Brazilian army and humanitarian organizations (IOM, 2021).

The mentioned church is the base of the “Orinoco project” established and funded by Caritas and the United States Agency for the International Development (USAID) and provides water, sanitation, food, and hygiene facilities for migrants. In addition, according to the United Nations International Organization for Migration (IOM), the bus station is an area with the capacity to shelter up to 900 people living in tents and offer them food, sanitation, and protection. At the time of the study, it was estimated that 1,603 people lived in the tent area, including 466 women (IOM, 2021, Soeiro et al., 2021a, Soeiro et al., 2021b). Many of those migrants are undocumented because they entered Brazil after the government officially closed the border due to the COVID-19 pandemic. Some with documents were living in those conditions because they were awaiting vacancies in the official shelters managed by UNHCR or were waiting to be called to work elsewhere in Brazil.

Among the estimated number of 466 women living at the bus station, we received 197 filled questionnaires and we discard 20 because they were returned in blank, consequently we are reporting data from 177 women. If we take into account the number of adolescents included in previous reports (n=167) (Soeiro et al., 2021a, Soeiro et al., 2021b) we were able to reach 73.8% of the women living outside the official shelters. The sociodemographic characteristics of the participants are presented in Table 1. The mean age (± SD) of the women was 28.0 ± 6.8 years (range 18–49), 69 self-identified as biracial, and 67.2% had migrated within the year 2020. Table 2 outlines some reproductive characteristics of the participants. We observed that 57 women reported a history of more than three children, 68 women with children reported at least one unplanned pregnancy, and 52.5% of the women intended to become pregnant in the near future.

**Table 1:** Sociodemographic characteristics of Venezuelan migrant women, Roraima, Brazil (n=177).

Variables		n (%)
*Age (years)*	≤19	20 (11.3)
	20 – 29	89 (50.3)
	30 – 39	54 (30.5)
	40 – 49	14 (7.9)
Ethnicity*	Biracial	69 (39.0)
	Black	42 (23.7)
	White	39 (22.0)
	Asian	22 (12.4)
	Indigenous	5 (2.8)
*Cohabitation status*	With a partner	120 (67.8)
	Without partner	51 (28.8)
	Decline to answer	6 (3.4)
*Schooling (years*)	Illiterate	1 (0.6)
	1-7	6 (3.4)
	8 – 12	88 (49.7)
	≥ 13	72 (40.7)
	Decline to answer	10 (5.7)
*Local of shelter*	Homeless (bus station)	116 (65.5)
	UN shelter	38 (21.5)
	Rented house or room	23 (13.0)
*Year in which migrated to Brazil*	2016	1 (0.6)
	2017	3 (1.7)
	2018	58 (32.8)5)
	2019	18 (10.2)
	2020	89 (50.3)
	Don´t remember	8 (4.5)
Reasons reported why migrated**	Lack of economic opportunities	143 (80.8)
	Social insecurity	23 (13.0)
	Corruption	7 (4.0)
	Medical assistance	2 (1.1)
	Refuse to answer	2 (1.1)

*Self-reported according to the Brazilian Institute of Geography and Statistics; **More than one answer allowed.

**Table 2:** Questions about reproductive health of Venezuelan migrant women, Roraima, Brazil (n=177).

Reproductive health questions		n (%)
*Have you ever been pregnant?*	Yes	150 (84.8)
	No	27 (15.3)
*How many of children do you have?*	0	27 (15.3)
	1-2	92 (52.0)
	3-4	46 (26.0)
	5 or more	11 (6.2)
	No answer	1 (0.6)
*Did you plan your previous pregnancies?*	No	68 (38.4)
	Yes	85 (48.0)
	Not applicable	24 (13.6)
*Do you have intention to have children in the future?*	No	76 (42.9)
	Yes	101 (57.1)

Data from access and use of the SRH services are shown in Table 3. A total of 134 women reported visiting a healthcare facility looking for gynecological attention, 115 sought SRH services, and 78 reported that they were satisfied with the service. However, 33/177 women were not aware of any healthcare service that offers SRH attention. Furthermore, the main reasons women reported seeking healthcare after migration were contraception, reported by 40 women, followed by antenatal care (ANC), reported by 30 women. Regarding the 40 women who sought a contraceptive method, 16 reported not being able to receive the method of their choice and 15 stated that the subdermal implant was the contraceptive they were looking for.

**Table 3:** Access and use of the sexual and reproductive health services reported by Venezuelan migrant women, Roraima, Brazil (n=177).

		N (%)
*Have you been to any healthcare facility?*	Yes	115 (65.7)
	No	62 (34.3)
For what reason?*	Contraception	40 (34.8)
	Antenatal care	30 (26.1)
	Postnatal care	1 (0.7)
	Gynecological problem	19 (16.5)
	Gender based violence	4 (3.5)
	Other health attention	21 (18.3)
*For those looking for contraception, did you obtain what do you wished? (n=40)*	Yes	26 (65.0)
	No	14 (35.0)
*If you did not obtain the contraceptive that you wish, do you obtain any other? (n-14)*	Yes	2 (14.3)
	No	12 (85.7)
*Which contraceptive are looking for? (n-40)*	Cooper IUD	2 (5.0)
	LNG-IUD	7 (17.5)
	Subdermal implant	13 (32.5)
	Injectables	11 (27.5)
	Tubal ligation	5 (12.5)
	Oral contraception	2 (5)
*Are you happy with the attention received at the healthcare facility? (n=115)*	Yes	96 (83.5)
	No	19 (16.5)
*Are you satisfied with the SRH´s attention since you migrated to Brazil? (n=115*)	Satisfied	78 (67.8)
	Partially satisfied	20 (17.4)
	No satisfied	6 (5.2)

*More than reason allowed.

Regarding the data collected on menstrual period and hygiene, the women reported challenges accessing supplies. We found that 110 of the participating women reported that menstruation is a normal event in their life; however, 39 referred to it as a painful or horrible event. In Table 4 we presented questions made about access and use of menstrual materials and only 54 reported that the menstrual hygiene pads they received were comfortable and 64 of the women reported that the hygiene products were not enough for their needs. In addition, most women mentioned that they are afraid that someone is watching or even worried that someone might attack them while they are changing their absorbents or pads. Furthermore, about a third of them say they never have a suitable place to change their absorbents or pads and almost 100% of the participating women reported that they did not want to use reusable products for menstrual hygiene.

**Table 4:** Information about access and use of menstrual material (MM) among Venezuelan migrant women, Brazil (n=177).

Questions	Answers (n)				
	always	often	sometimes	never	missing answer
-Do you have a suitable place to store your MM (if there is any) until your next menstruation?	93	16	31	20	17
-Are you worried that other people would see your used MM in the place where you discarded it?	116	7	21	19	14
-Can you immediately discard your used MM the way you would want to?	96	18	35	17	11
-Can you immediately discard your used MM?	105	15	35	12	10
-Can you wash your hands whenever you want to?	108	9	44	5	11
-Are you worried that other things will hurt you when you change your MM (animals, insects, etc.)?	86	12	27	38	14
-Are you worried that someone would hurt or attack you when you change your MM?	82	6	32	46	11
-Are you worried that someone would see you when you change your MM?	105	36	5	20	11
-Do you have a suitable place to change your MM?	60	4	52	51	10
-Do you feel comfortable carrying the MM with you?	104	12	34	19	8
-Are you worried that the MM you use will allow the blood to leak out through your clothes?	61	22	53	34	7
-Are you able to get more MM during your menstrual period if you need it?	46	24	53	54	0
-Have you received enough MM in order to change it with the desired frequency?	39	17	51	64	6
-Are the MM you receive comfortable?	54	23	56	36	8

MM: menstrual material

## 4 Discussion

Our study showed that the interviewed women were young, mainly they migrated in search of better economic opportunities, and most of them lived in precarious situations in tents provided by the Brazilian army, which is analogous to living on the streets. Further, our results indicated a serious failure to assure resources to guarantee reproductive justice to this group of migrant women. Also indicated that although the government closed the border with Venezuela on March 2020 people still entered Brazil by alternative routes, mainly because there is no physical barrier between the countries (IOM, 2020), which increased the number of persons looking for medical attention and challenged the access of migrants women to SRH services and care. Although Brazilian law guarantees access free of cost to all persons who are in the national territory without distinction between nationals and migrants (legal or non-legal), in fact, the system requires identification from the National Health Service, and many migrants do not know the bureaucratic process to obtain this document, which is a barrier to receiving attention at health services (Brasil. Ministério da Saúde 2009).

The situation of the migrant women in the Brazilian Amazon region regarding access to SRH care is not different from that previously described by our group which was aggravated by the COVID-19 pandemic (Bahamondes *et al*., 2020; Makuch et al., 2021a, Makuch et al., 2021b; Soeiro et al., 2021a, Soeiro et al., 2021b). In addition to migration problems, migrant women of reproductive age are vulnerable due to gender inequalities, including unplanned pregnancy due to lack of access to or availability of contraceptive methods (Olsen *et al*., 2021). We found that women were unable to obtain the contraceptive method they wanted to use, and in many cases, they were unable to obtain any method at the public service network. Although Cu-IUD is available at the Brazilian public health care network, many women were unable to obtain it because there are no health care professionals trained in insertion and removals (Bahamondes *et al*., 2020; Makuch *et al.*, 2021a). Hormonal-IUD and contraceptive implants are not available in Brazil in the public sector, and the latter was the method with high procurement by migrant women (Makuch *et al.*, 2021a).

For migrant women who live in precarious situations, it is important to avoid an unplanned pregnancy, and the use of any long-acting reversible contraceptive (LARC) method could be an important advantage. Further, many of the interviewed women reported that they want to have a child in the near future, probably when their living conditions improved in other parts of the Brazilian territory. However, the lack of access to contraceptive methods, is not only a problem for Venezuelan migrant women because it is also common among the nationals (Gragnolati *et al*., 2013; Olsen *et al*., 2021). Furthermore, almost 20% of the women reported not being familiar with any healthcare service; however, among those who received attention, almost two thirds of them were satisfied with the service received. On one hand, we could consider the attention given at the Brazilian public health service generally good on a national level; on the other hand, we need to consider a courtesy bias from the respondents.

Our results indicated that the healthcare system in Roraima was not prepared after a nearly five year increase in the number of persons who required attention due to the migration crisis (Makuch *et al*., 2021a) and was certainly impacted again due to the COVID-19 pandemic. Many SRH services, including contraceptive provision, were closed around the country because they were considered non-essential services (Bahamondes *et al*., 2022). Due to the COVID-19 pandemic, many health care providers were reallocated to attend COVID-9 cases (Bahamondes and Makuch, 2020; Makuch *et al*., 2021b; Bahamondes *et al*., 2022), many resigned, and the state faced a lack of supplies, medicines, and equipment, including ventilators.

Regarding period poverty and the availability of menstrual hygiene products, our results agreed with a previous report with Venezuelan adolescent migrants (Soeiro *et al*., 2021a) in which women reported a lack of access to hygiene products and precarious sanitary conditions, a cruelty situation that exposes women to gynecological infections. Why the women need to claim about menstrual hygiene products if the cost of these products in the general budget of the either federal, state or municipal governments and the humanitarian organizations is a neglected issue. In Brazil, a kit with two reusable feminine menstrual cups is US$ 10. However, we must take into account that the participating women reported that they did not want to use reusable products for menstrual hygiene, although we failed to ask specifically about menstrual cups. It is possible that the women refuse to use non-disposable menstrual hygiene products due to the difficulties accessing a sanitation service with water on demand. It was stated that women managed menstrual periods differently when they were at home vs. away from home. Women at home have the opportunity to dispose of menstrual products in the waste; however, when they are away from home, they need clean public toilets with privacy (Kaur *et al*., 2018).

One explanation for why there is no more proactive action on this issue could be that the Brazilian government does not have a policy to address period poverty. In October 2021 the Brazilian president used the power of the veto to defeat a bill that obligated the government to provide menstrual hygiene products to underprivileged and vulnerable women. There is a need for care and dignity, and menstrual hygiene products are a very important part of a woman's life, to make the precarious life of these migrant women a little better and give them a decent quality of daily living.

Our study presents limitations and strengths. One of the limitations was the lack of opportunity to interview women at the UNHCR official shelters due to the COVID-19 pandemic; however, we could also consider this as strength because the reality of migrant women who live in precarious conditions could be similar to that of those observed in other countries (Rivillas-García et al., 2020). The main limitation regarding our evaluation of period poverty is similar to previous research that are limited to describing knowledge, attitudes, and practices about menstrual hygiene and access to menstrual products, with no further implementation of actions to improve or change these menstrual hygiene practices (Kuhlmann et al., 2017). However, the strength of our study was to interviewed adult women who living outside the official shelters which complement previous studies conducted in the north of Brazil (Bahamondes *et al*., 2020; Makuch et al., 2021a, Makuch et al., 2021b; Soeiro et al., 2021a, Soeiro et al., 2021b).

The prevention of unplanned pregnancy is crucial among migrant women because they arrive in a new country without employment opportunities, and an unplanned pregnancy could end in an unsafe abortion and even in maternal morbidity and mortality (Fundo de População das Nações Unidas 2018). Because it is convenient for displaced women to not have to perform daily actions, such as taking the pill, LARC methods increase the effectiveness of contraception. Menstrual poverty is a result of women's lack of access to resources, infrastructure, and menstruation care knowledge. Moreover, feminine hygiene products are not accessible to them, and they do not have both treated water and a safe place to care for intimate hygiene themselves. This phenomenon is directly linked to social, economic, and gender inequalities (Bahamondes and Makuch, 2020).

## 5 Conclusion

The vulnerabilities of this cohort of Venezuelan migrant women in Brazil who lived mainly out of the official shelters further increase when they struggle with no knowledge of how to access SRH services, lack of provision of LARC methods, risk of unplanned pregnancy, and inappropriate access to menstrual hygiene products and sanitary services. There are several challenges to be overcome to ensure SRH care for migrant women in Brazil.

## Funding

This work was funded by the CEMICAMP, Center for Research in Reproductive Health of Campinas.

LR and NN received funding from SRH, part of the UNDP-UNFPA-UNICEFWHO-World Bank Special Programme of Research, Development and Research Training in Human Reproduction (HRP), a cosponsored programme executed by the World Health Organization (WHO), to complete their MSc degree.

## Authorship contribution statement

LR, RS, LB and MLC had the initial idea for the study. LR and RES were responsible for data collection. All authors were responsible for planning the analysis and interpretation of data. LR, NNGM, FS, and RES wrote the first draft of the paper. All authors read and approved the final manuscript.

## Declaration of Competing Interest

The authors have declared that no competing interests exist.
